# Urinary incontinence 6 weeks to 1 year post-partum: prevalence, experience of bother, beliefs, and help-seeking behavior

**DOI:** 10.1007/s00192-020-04644-3

**Published:** 2021-01-23

**Authors:** Heidi F. A. Moossdorff-Steinhauser, Bary C. M. Berghmans, Marc E. A. Spaanderman, Esther M. J. Bols

**Affiliations:** 1 Care and Public Health Research Institute (CAPHRI), Maastricht University, P.O. Box 616, 6200 MD Maastricht, The Netherlands; 2 Pelvic Care Unit Maastricht, CAPHRI, Maastricht University Medical Centre (MUMC+), Maastricht, The Netherlands; 3 Department of Obstetrics and Gynaecology, MUMC+, Maastricht, The Netherlands

**Keywords:** Help-seeking, Pelvic floor muscle exercises, Post-partum, Prevalence, Quality of life, Urinary incontinence

## Abstract

**Introduction and hypothesis:**

Post-partum, women often experience urinary incontinence (UI). However, the association between experienced UI bother and UI beliefs and help-seeking behavior is less known. Therefore, we aim to investigate the prevalence of self-reported UI, the level of experienced bother and beliefs, to explain help-seeking behavior for UI in women in the Netherlands from 6 weeks to one year post-partum.

**Methods:**

A digital survey among post-partum women, shared on social media, was used for recruitment. The survey consists of: 1. demographic variables, 2. International Consultation on Incontinence Questionnaire-Urinary Incontinence Short Form (ICIQ-UI SF), 3. ICIQ Lower Urinary Tract Symptoms Quality of Life (ICIQ-LUTSqol), and 4. questions on beliefs and help-seeking behavior. For analysis, descriptive statistics and the independent samples t-test were used to determine differences between help- and non-help-seekers.

**Results:**

415 women filled in the survey. The mean age was 30.6 years (SD 4.0, range 21–40) of which 48.2% was primiparous. The overall prevalence of UI was 57.1% (95% confidence interval (CI) (52.3–61.8)). Primiparous women reported a statistically significantly lower overall prevalence than multiparous women, 52.0% and 61.9% respectively (*p* = .043). UI was reported as bothersome in 38% of women, 25% of all women sought help. Help-seeking women showed significantly higher scores for bother, measured by the ICIQ-UI SF, than non-help seekers (*p* = .001).

**Conclusions:**

More than half of all post-partum women in the Netherlands from 6 weeks to one year post childbirth experience UI (57.1%), 38% classified their UI as bothersome. In total 25% of UI women sought professional help.

## Introduction

Urinary incontinence (UI) as a symptom is defined by the International Continence Society as the ‘complaint of involuntary loss of urine’ [1]. Prevalence numbers of UI from six weeks to one year post-partum range from 10.5 to 63.0% [2, 3]. The wide range in reported prevalence might be explained by the use of different case definitions, post-partum period and study methodology. On the one hand, the International Consultation on Incontinence (ICI) has recommended to accompany prevalence numbers with experienced symptom bother, and on the other hand to measure this construct with high quality measurement instruments preferably within the International Consultation on Incontinence Questionnaire (ICIQ) structure [4]. Despite the ICI recommendations, symptom bother is often not included in prevalence studies. Moreover, a variety of measurement instruments are used for symptom bother, ranging from high quality to non-validated self-constructed questionnaires [2, 3]. These factors influence reliable prevalence numbers for (bothersome) UI, which are of relevance for health care providers, policy makers, and researchers [5]. To date, knowledge on crude prevalence numbers (categorized by type of UI, post-partum period, or parity) and symptom bother measured with measurement instruments within the ICIQ structure in the post-partum period are largely lacking.

The level of bother, type and severity of UI are associated factors in help-seeking behavior in the general female population [6]. After delivery, women often believe that their UI will improve by itself [7]. Pelvic floor muscle training (PFMT) is an effective treatment option for post-partum women with UI and recommended as first treatment option in guidelines on UI [8]. However, to our knowledge it is unknown if and what kind of experiences and daily activities contribute to help-seeking and why post-partum women do not seek help. Therefore, the aim is to investigate the prevalence of self-reported UI, the level of experienced bother and beliefs, to explain help-seeking behavior in women in the Netherlands from 6 weeks to one year post-partum.

## Material and methods

### Study design

A cross-sectional design was used to describe the prevalence, bother, believes, and help-seeking behavior of post-partum women. The Medical Ethics Committee of the Maastricht University Medical Centre (MUMC+) approved this study (number 2019–1320). All women of 18 years and older, regardless of parity and between 6 weeks and one year post-partum, who were able to fill in a digital questionnaire in the Dutch language were eligible to participate. Based on an overall prevalence of UI in women of 33%, a Z statistic of 1.96 and precision of 0.05, a minimal sample size of 340 women was estimated to fill out the survey [9]. Nationwide midwifery and physical therapy practices were among others asked to share a social media message (using Facebook and LinkedIn), containing brief information on the study (goal, eligibility) and a link to the patient information letter and digital survey. In this context a physical therapist is defined as a physical therapist, educated and specialized in health problems related to the pelvic floor and organs in the pelvis minor.

Before proceeding to the anonymized digital survey, eligible women signed informed consent electronically, in agreement with ethical regulations. The survey took 10 to 15 min to complete.

### Outcome measures

The survey consists of four parts: 1. demographic variables like age, educational level and parity, 2. International Consultation on Incontinence Questionnaire Short Form (ICIQ-UI SF) [10], 3. International Consultation on Incontinence Questionnaire Lower Urinary Tract Symptoms Quality of Life (ICIQ-LUTSqol) [11] and 4. questions on beliefs and help-seeking behavior regarding UI.

The ICIQ-UI SF consists of four questions and provides an indication of UI severity. The first question is with regard to the frequency of UI, with a score of 0 (never losing urine) to 5 (losing urine all the time). The second question asks for amount of urine loss, with four response categories ranging from 0 (no loss) to 6 (large amount). The third question evaluates impact of UI on daily life, ranging from 0 (not at all) and 10 (a great deal). The total score ranges from 0 (no UI) to 21 (very severe problem). The total score is divided into four categories: slight (1–5), moderate (6–12), severe (13–18), and very severe (19–21) [12]. A fourth question on the occurrence of symptoms of UI was used to indicate SUI, UUI and MUI [13]. A participant was considered to have SUI when leaking urine with a cough or a sneeze and/or when physically active/exercising, but not before getting to the toilet. UUI is considered when the respondent leaks before getting to the toilet. A respondent with MUI experiences both SUI and UUI.

The ICIQ-LUTSqol is a condition-specific health-related quality of life questionnaire (20 questions), adapted for use within the ICIQ structure from the King’s Health Questionnaire [11]. It contains 19 questions that can be scored on life restrictions, emotional aspects and preventive measures. It is scored on a four-point Likert scale ranging from 1 (not at all) to 4 (a lot). Three questions on relationships, sex life, and family life include additionally ‘not applicable’. ‘Not applicable’ was considered as not affecting daily life. The sum score ranges between 19 and 76. A higher score indicates a higher impact on quality of life. Every question is accompanied by a question regarding experienced bother (ranging from 0 (no bother) to 10 (extreme bother)). It is arbitrarily decided that a score of at least 5 indicates significant bother on a specific item. The 20th question is on how much urinary symptoms interfere with daily life, scored between 0 to 10 (like bother). Both the ICIQ-UI SF and ICIQ-LUTSqol are rated as ‘high quality’ questionnaires and are recommended by the ICI [4].

All respondents at least filled in the demographic variables and ICIQ-UI SF. Answering ‘never losing urine’ at the frequency item of the ICQ-UI SF indicated continence and consequently the survey was finished. When reporting UI, women completed the remaining two parts on quality of life and help-seeking behavior.

The questions on beliefs and help-seeking behavior were self-constructed. Selection of question and answer options was based on models explaining help-seeking behavior, discussion with experts in the field (epidemiologists and obstetrician/gynecologist) and modified accordingly [14]. Moreover, questions were reviewed by an expert for readability and comprehensiveness, followed by field testing. Ultimately, six questions were developed including four topics on health seeking behavior (actual help-seeking, reason(s) to (not) seek help, reason to seek help in the future and consulted health-care provider(s)) and two topics on beliefs (self-perceived prognosis and self-perceived best intervention to treat UI in general).

### Data analysis

Data was analyzed using descriptive statistics presented as proportions (frequency and means (SD)) and correlation was performed by Pearson’s correlation coefficient. Post-partum women were categorized into three groups: 6 weeks to 3 months, 3 to 6 months and 6 to 12 months post-partum. Independent sample t-tests were conducted to compare help-seekers and non-help seekers with regard to UI severity (ICIQ-UI SF total score), bother (ICIQ-LUTSqol total score), and interference in daily life. Chi-square tests were used to test relationships between categorical variables. One-way analysis of variance (ANOVA) was used to explore differences in experienced bother, measured with the ICIQ-UI SF scores, across the three post-partum periods. The effect size was estimated with Cohen’s *d*. Cohen’s *d* presents the difference between groups (help-seekers and non-help-seekers) in standard deviation units. To interpret the strength of the effect size we followed the guidelines proposed by Cohen: .2 = small, .5 = medium, .8 = large. An alpha of 0.05 is considered statistically significant. Analyses were done using IBM Statistical Package for Social Sciences (SPSS) version 26.0 (New York, NY, USA).

## Results

In March 2020, 415 women filled in the survey. The mean age was 30.6 years (SD 4.0, range 21–40) of which 48.2% (200/415) was primiparous (Table 1). A total of 37.7% (157/415) followed secondary and 61.4% (255/415) tertiary education. The overall prevalence of UI was 57.1% (95% confidence interval (CI) 52.3–61.8). Primiparous women reported a lower overall prevalence of UI compared to multiparous women (52%, 104/200) and 61.9%, 133/215 respectively) which was statistically significant (*p* = .043). The prevalence of UI does not change significantly across the three post-partum periods (*p* = .15). However, the pattern over time shows the highest prevalence between 6 weeks and 3 months with 66.7% (50/75), almost statistically significant decreasing to 52.6% (61/116) between 3 and 6 months after which there is no significant change thereafter (56.3% (126/224), between 6 and 12 months. SUI (62.9%, 149/237) was the most frequently reported type of UI.

**Table 1:** Characteristics of post-partum women, urinary incontinence prevalence, and ICIQ-UI SF questionnaire results

		Overall (*n* = 415)	6 weeks – 3 months (*n* = 75)	3 months – 6 months (*n* = 116)	6 months – 12 months (*n* = 224)
Age	Mean (SD, range)	30.6 (4.0, 21–40)	30.7 (4.0, 23–40)	30.1 (4.0, 21–40)	30.8 (4.0, 21–40)
Prevalence	Total	237 (57.1) 95% CI: 52.3–61.8	50 (66.7) 95% CI: 56.5–77.4	61 (52.6) 95% CI: 43.3–61.8	126 (56.3) 95% CI 49.3–63.3
	SUI	149 (62.9)	25 (50.0)	38 (62.3)	86 (68.3)
	UUI	23 (9.7)	7 (14.0)	4 (6.6)	12 (9.5)
	MUI	47 (19.8)	10 (20.0)	14 (23.0)	23 (18.3)
	Other (such as UI during sleep or UI for no obvious reason)	18 (7.6)	8 (16.0)	5 (8.2)	5 (4.0)
ICIQ-UI SF	Total score (0–21) Mean (SD, range)	8.1 (3.4, 3–17)	8.4 (3.6, 3–16)	7.4 (3.4, 3–16)	8.3 (3.4, 3–17)
Frequency	About once a week or less often	104 (43.9)	19 (38.0)	32 (52.5)	53 (42.1)
	Two or three times a week	59 (24.9)	10 (20.0)	14 (22.9)	35 (27.7)
	About once a day	31 (13.1)	12 (24.0)	5 (8.2)	14 (11.1)
	Several times a day	40 (16.9)	9 (18.0)	9 (14.8)	22 (17.5)
	All the time	3 (1.2)	0 (0)	1 (1.6)	2 (1.6)
Amount	A small amount	212 (89.5)	43 (86.0)	55 (90.2)	114 (90.5)
	A moderate amount	23 (10.1)	7 (14.0)	6 (9.8)	11 (8.7)
	A large amount	1 (0.4)	0 (0)	0 (0)	1 (0.8)
Overall Interference (range 0–10)	≥5	90 (38.0)	22 (44.0)	19 (31.1)	49 (38.9)
Categories (1 missing)	Slight (1–5)	70 (29.7)	16 (32.0)	21 (34.4)	33 (26.2)
	Moderate (6–12)	136 (57.6)	27 (54.0)	35 (57.4)	74 (58.7)
	Severe (13–18)	30 (12.7)	6 (12.0)	5 (8.2)	19 (15.1)
	Very severe (19–21)	0 (0)	0 (0)	0 (0)	0 (0)

N = number, % = percentage, SD = standard deviation, CI = confidence interval, UI = urinary incontinence, SUI = stress urinary incontinence, UUI = urgency urinary incontinence, MUI = mixed urinary incontinence, ICIQ-UI-SF = International Consultation on Incontinence Questionnaire Urinary Incontinence Short Form

UI frequency of once a week or less was reported in 43.9% (104/237) and in 89.5% (212/237) of the cases it was a small amount of urine (Table 1). The impact of UI based on the ICIQ-UI SF score was reported by 29.7% (70/236) of the women as slight and by 57.6% (136/236) as moderate. There were no statistically significant differences for the ICIQ-UI SF score across the three post-partum periods (*p* = .06). The mean interference in daily life based on ICIQ-UI SF was 3.8 (SD 2.4, range 0–9), whereas 38% (90/237) of the respondents reported an overall interference in daily life of ≥5. The mean ICIQ-LUTSqol was 29.8 (SD 7.9, range 20–58). Respondents reported that they experienced a significant bother on three daily activities based on the ICIQ-LUTSqol. The first is on ‘physical activities’, like going for a walk, run or sports. The second is regarding the ‘need to change wet underclothes’ and the third is about ‘worrying because of smell’. Respondents with UI were least affected and bothered by the items on maintaining friendships, the effect on sleep and feeling tired. The correlation between the total score of the ICIQ-UI SF and the ICIQ-LUTSqol was high (0.74, *p* = .001, R^2^ = 0.54).

In total, 25.7% (61/237) of the respondents sought help for their UI post-partum (Table 2). The majority of women seeking help (92%) visited a physical therapist. The reasons provided for not seeking help were: minimal bother (52.9%, 91/172) and the idea that their UI would improve in time by itself (54.1%, 93/172). The most important reasons for seeking help in the future were: the constant use of pads (45.9%, 79/172), leaking/getting wet clothes (35.5%, 61/172), the feeling that others can smell the urine loss (27.9%, 48/172) or hindrance at work (27.9%, 48/172). With regard to seeking help in the future, 32% (55/172) of women reported one and 68% (117/172) reported two or three reasons why they would seek help in the future.

**Table 2:** Beliefs and help-seeking behavior in relation to urinary incontinence

**BELIEFS**		
**Prognosis UI without seeking help**	**Help-seekers (*N*** **= 61)**	**Non-help-seekers (*N*** **= 172)**
Complete recovery	2 (3.2)	38 (22.1)
Good improvement	2 (3.2)	47 (27.3)
Some improvement	12 (19.7)	34 (19.8)
About the same	17 (27.9)	41 (23.8)
Some deterioration	15 (24.6)	11 (6.4)
Great deterioration	9 (14.8)	1 (0.6)
Worse than ever	4 (6.6)	0 (0)
**Best way to solve UI**		
Surgery	3 (4.9)	3 (1.7)
Medication	2 (3.3)	0 (0)
Pelvic floor muscle exercises	46 (75.4)	143 (83.1)
It will resolve by itself	1 (1.6)	6 (3.5)
There is no solution	1 (1.6)	4 (2.3)
I do not know	6 (9.8)	13 (7.6)
Other	2 (3.3)	3 (1.7)
**HELP-SEEKING**	**Help-seekers**	**Non-help-seekers**
**Reason to seek help**	I sought help because^*^	**I will seek help in the future if**^**#**^
Getting wet clothes/leaking through	2 (3.3)	61 (35.5)
Need to use pad all the time	11 (18.0)	79 (45.9)
Others can smell me	1 (1.6)	48 (27.9)
Hindrance during sports	12 (19.7)	27 (15.7)
Hindrance during work	4 (6.6)	48 (27.9)
Hindrance playing with children	5 (8.2)	32 (18.6)
Hindrance during household tasks/activities	0 (0)	14 (8.1)
I do not know	24 (39.3)	25 (14.5)
Other reason(s)	0 (0)	10 (5.8)
**Reason not to seek help**		**Non-help-seekers (N = 172)**
Minimal bother		91 (52.9)
It will improve by itself		93 (54.1)
Lack of time		25 (15.5)
No childcare		13 (7.6)
Costs		7 (4.1)
No transport		3 (1.7)
Other		28 (16.3)

N = number, ^*^ = only one response option, ^#^ = multiple response options possible

**Fig. 1:**
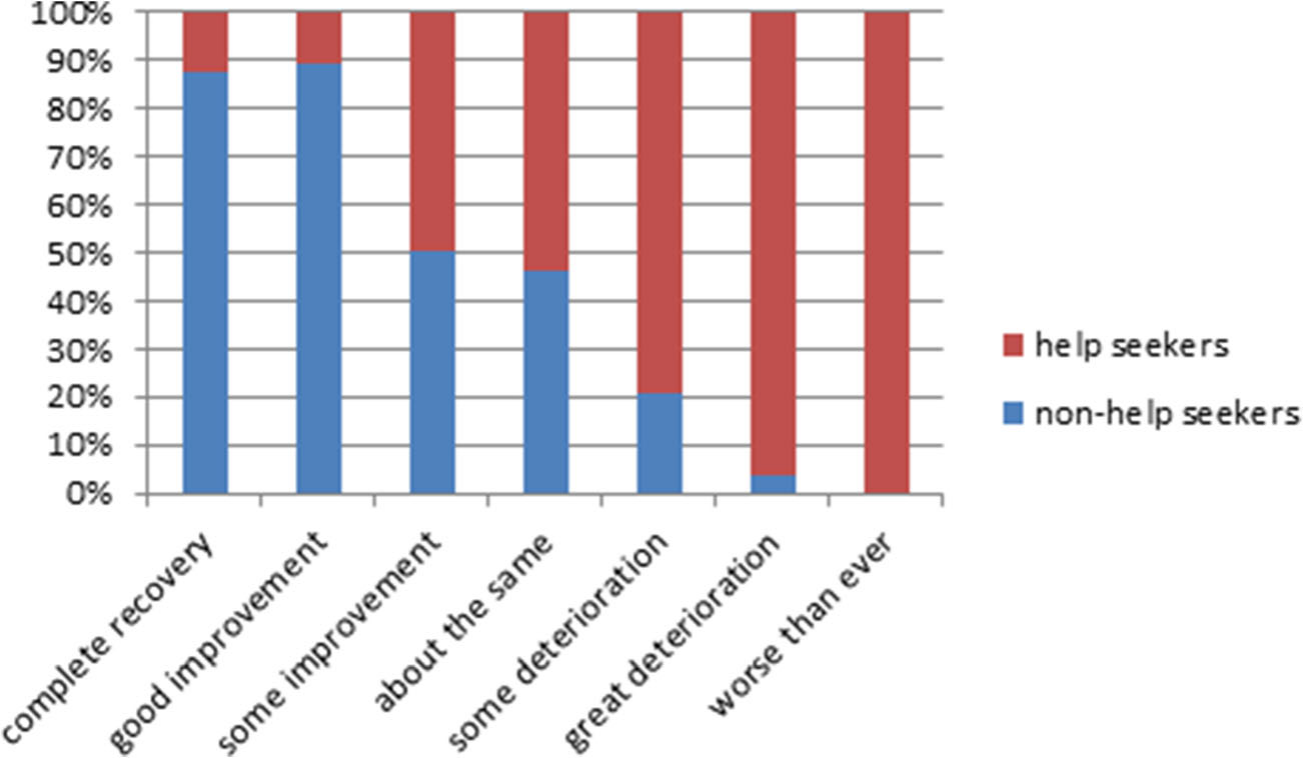
Beliefs about prognosis of urinary incontinence if help is not sought among non-help seekers and help seekers

More women, 49.4% (85/172) who did not seek help in contrast to 6.4% (4/61) of the women who did seek help for their UI, thought that their UI would completely resolve or improve a great deal in the future (*p* < .001). Figure 1 shows the beliefs about self-perceived prognosis of UI among non-help-seeking and help-seeking women as relative percentages of 100%. Of all women with UI, 79.7% (189/237) thought that the best way to treat their UI would be pelvic floor muscle exercises.

Help-seeking women showed significant higher scores than non-help-seeking women regarding ICIQ-UI SF (*p* = .001), ICIQ-LUTSqol (*p* < .001), and interference in daily life (*p* = .002), with corresponding medium effect sizes (ICIQ-UI SF total score: Cohen’s *d* = 0.52, ICIQ-LUTSqol total score: Cohen’s *d* = 0.57, and interference in daily life: Cohen’s d = 0.48). Parity, level of education, age, type of UI, ICIQ-UI SF Amount, and ICIQ-UI SF Frequency showed only weak correlations with help-seeking (ranging between 0.1–0.24).

## Discussion

### Principal findings

This study showed that the overall crude prevalence of self-reported UI post-partum is high (57.1%), with 38% experienced as bothersome UI. SUI is the most prevalent type (62.9%), followed by MUI (19.8%) and UUI (8.9%).

The high overall crude prevalence in this study is not uncommon compared to other studies [2]. The prevalence of UI in primiparous women was 52.0% rising to 61.9% in multiparous women. This is in line with other research, indicating that the first delivery is a major risk factor for UI [15]. The prevalence of UI post-partum did not change significantly in the course of the first year post-partum. Although the initial prevalence between 6 weeks and 3 months almost statistically significantly decreased at 3 to 6 months post-partum, the difference between this initial period and the second half of the year after childbirth was not statistically significant. Both Gartland et al. (2016) and Brown et al. (2015) reported a decrease of UI prevalence and thereafter an increase throughout the first year after childbirth [16, 17]. The decreasing prevalence at three to six months post-partum might be explained by physiological recovery and the rise thereafter because of an increase in return to activities provoking UI, such as physical activity or work [18].

This is one of the first studies to report the number and reasons of post-partum women to seek help for their UI [19]. In total 25.7% of post-partum women sought help for their UI, in 92% of cases they visited a physical therapist. This reflects the recommendations in the guidelines on UI for the general practitioners in The Netherlands proposing physical therapy as a first treatment option [20]. The fact that participants were recruited through social media from both midwifery and physical therapy practices this number might have been influenced. The help-seeking women reported a greater interference in daily life compared to not-help-seeking women. 46% of help-seeking women think that their UI would deteriorate when they would not seek help in contrast to 7% of non-help-seeking women (*p* < .001). This is in line with other studies in which women mentioned that they did not seek help because they were not greatly bothered by their UI and thought that it would diminish by itself in time [7]. However, up to 91% of women with SUI after their first delivery still report SUI 12 years later [21]. Although UI is not life threatening, women in the general population with UI report lower health-related quality of life and mental well-being and 45% of women report a moderately to totally limiting effect on exercise [22]. Women with UI in this study reported significant bother of UI regarding physical activities like in the study of Monz et al. [22]. Women with UI during physical activities adapt by e.g. reducing the intensity and avoiding specific UI provoking activities that may impact physical fitness and mental health [23].

### Clinical and research implications

Generally, women in the Western world have a final check at six to eight weeks post-partum by a midwife or gynecologist. This recovery period might be short to judge actual pelvic floor dysfunctions [24]. On the one hand, the contractility of the pelvic floor muscles are considered to need at least 12 weeks to recuperate and at four to six months post-partum the distensibility of the hiatal area is still significantly increased during Valsalva compared to early pregnancy which can limit the physical resilience of the pelvic floor [25]. On the other hand women are in the early post-partum period also busy finding a new balance in their life and their own health may be considered less important to them at that moment [7]. With this in mind it might be more appropriate to check the mother’s health regarding pelvic floor dysfunctions like UI at a later stage (three to six months post-partum). At the moment there is no validated easy assessment tool that evaluates women’s well-being in a broader, more general perspective. Therefore, an evidence-based selection tool investigating and mapping women’s health in general and the pelvic floor specifically, aiming to record whether and to what extent an intervention is warrant. For this purpose, aphysical therapist, as an expert on both women’s health and in conservative management of pelvic floor dysfunctions, may use the tool of the physical therapeutic diagnostic consultation that, given the medical diagnosis of UI, looks at the consequences of this health problem on three different levels, being the local level (impairments), personal level (disabilities) and the sociocultural level (restriction in participation [26]. Our results show that 75.4% of help seeking and 83.1% of non-help seeking women think PFMT is the best way to solve UI. This suggests that PFMT is a well-known treatment option in The Netherlands. However, this number might not reflect the knowledge of PFMT in other parts of the world as Asia and Africa. For example, 55.5% and 58% of pregnant women in Thailand and Malaysia, respectively, possessed knowledge of PFMT [27, 28].

### Strengths and limitations

The strength of this study is the large nationwide sample on post-partum women in The Netherlands. Another strength is the use of high quality, recommended questionnaires to measure the prevalence and bother of UI, and impact on quality of life. To our knowledge this is the first study to use the ICIQ-LUTSqol to study quality of life and therefor to evaluate bother extensively in post-partum women from 6 weeks to 1 year post-delivery, next to their relations with help-seeking behavior.

This survey has several limitations. Firstly, women in The Netherlands who do not speak Dutch could not fill in the survey. This might have influenced the outcome regarding the knowledge on the best treatment option for UI. Nevertheless, non-native speakers are less likely to be familiar with possible treatments e.g. PFMT [29]. Secondly, we did not ask if UI occurred before the first pregnancy or during the pregnancies. Therefore, we do not know at what stage in their obstetric history women experienced new onset UI. The third limitation comprises the possible risk of bias due to the accessibility of social media for recruitment. Though, in 2020, 75% of The Dutch population use Facebook and 38% LinkedIn [30]. Finally, the non-response rate is not known.

## Conclusion

More than half of all post-partum women in the Netherlands from 6 weeks to one-year post childbirth experience UI (57.1%), of whom, 38% classified their UI as bothersome.. In women with UI, 25% sought help and in 92% of cases this was with a specialized (pelvic) physical therapist. Help-seeking women experience higher impact on bother than non-help seekers.
